# Relevance of Trehalose in Pathogenicity: Some General Rules, Yet Many Exceptions

**DOI:** 10.1371/journal.ppat.1003447

**Published:** 2013-08-15

**Authors:** Hélène Tournu, Alessandro Fiori, Patrick Van Dijck

**Affiliations:** 1 Department of Molecular Microbiology, VIB, Leuven, Belgium; 2 Laboratory of Molecular Cell Biology, Katholieke Universiteit Leuven, Leuven, Belgium; The University of North Carolina at Chapel Hill, United States of America

## Trehalose: A Supporting Act More than a Starring Role

Trehalose, a natural disaccharide, consists of two glucose molecules linked by an α, α-1,1-glucoside bond. Apart from its function as a reserve carbohydrate, trehalose is known for its role as a stress protectant in many organisms across kingdoms. It is present in plants, invertebrates, fungi, and prokaryotes, but not in mammals. Despite its well-known protective roles against desiccation, freezing, starvation, and osmotic stress [1], trehalose has escaped categorization into a specific biological pathway. Interestingly, a role for its metabolism is emerging in the establishment of virulence traits in distantly related microbial species.

In general, although notable exceptions exist, the absence of a complete trehalose metabolism apparatus is associated with a lower pathogenic potential. The mechanisms involved, however, are less clear. Pathogens have engineered several distinct trehalose-associated mechanisms that contribute to cell morphogenesis, cell wall integrity, regulation of metabolism, and evasion from the host immune response.

## Trehalose Synthesis Pathways and Virulence: *E Pluribus Unum*?

**Figure 1:**
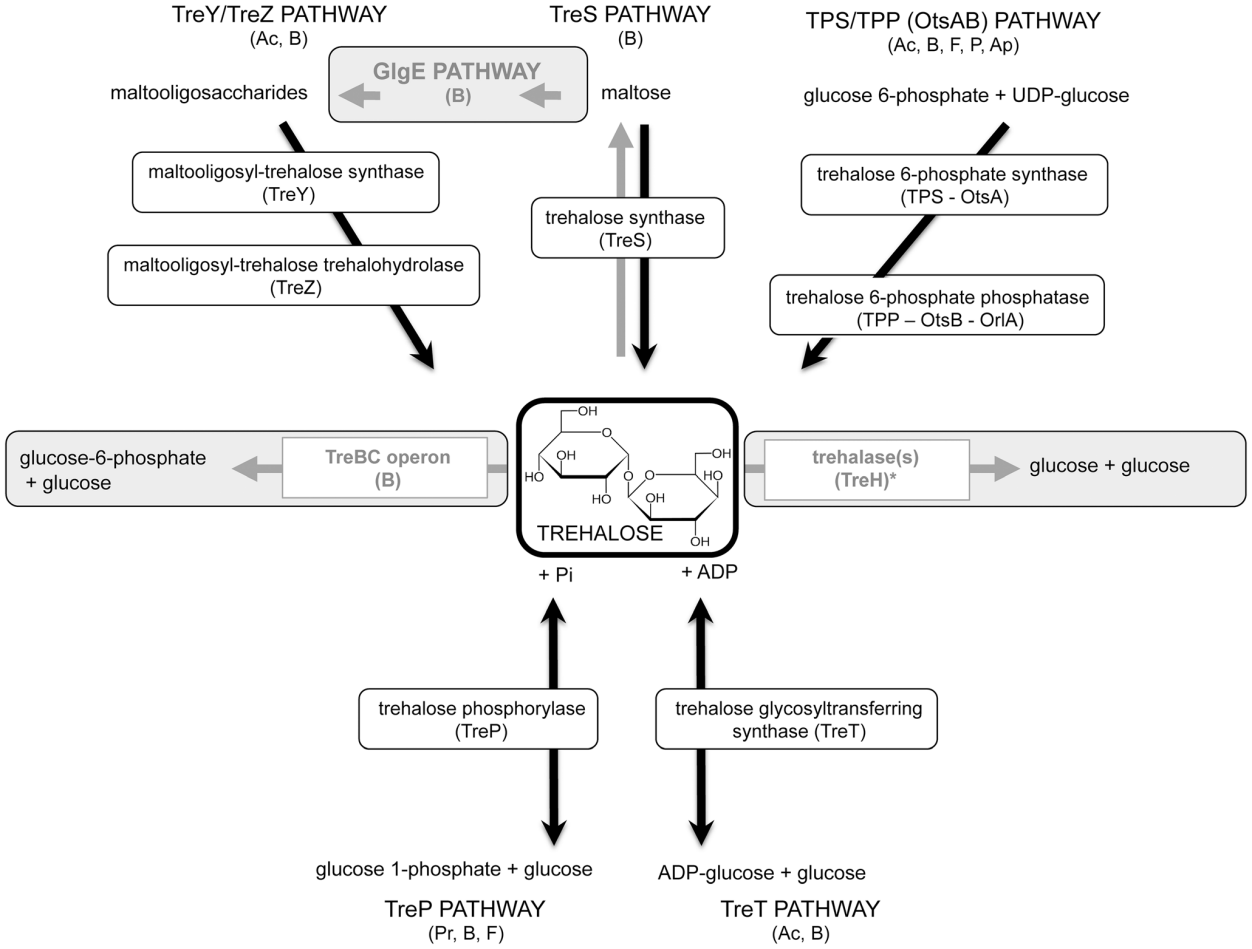
Trehalose metabolic pathways. Pathways of trehalose synthesis are highlighted in black, and pathways of degradation in grey. Major conserved enzymes are included. The TPS/TPP (OtsAB) is a two-step pathway. Glucose 6-phosphate and UDP-glucose are converted into trehalose 6-phosphate (T6P) by the trehalose 6-phosphate synthase enzymes. T6P is then converted into trehalose by the T6P phosphatase enzymes. The TreYZ pathway yields trehalose from glucans. The first step, operated by TreY (maltooligosyltrehalose synthase), mediates the inversion of the reducing-end glucosyl residue of α-1,4 glucan into α, α-1,1-linked non-reducing trehalosyl disaccharide end. This is followed by the cleavage of free trehalose from the glucan chain by TreZ enzyme (maltooligosyltrehalose trehalohydrolase). The TreS pathway consists of the trehalose synthase enzyme (TreS), and converts trehalose into maltose and vice versa. The TreS and the GlgE pathways are linked by the Pep2 enzyme, a maltokinase, that converts maltose into maltose 1-phosphate. The novel GlgE pathway converts trehalose via the TreS pathway into glucans and glycogen in *Mycobacterium tuberculosis* [33]. From maltose 1-phosphate, the essential maltosyl-transferase GlgE extends glucan chains, and the essential GlgB enzyme introduces α-1,6-linked branches to linear glucans. The TreBC operon consists of TreB, the trehalose PTS permease, which transports extracellular trehalose inside the cells and converts it into T6P. T6P is then converted into glucose 6-phosphate and glucose by TreC enzymes encoding T6P hydrolases. Trehalase enzymes degrade trehalose into two molecules of glucose, and have been categorized as cytoplasmic (or neutral) and extracellular (or acid) enzymes. The role of the TreP and TreT pathways in pathogenicity has not been demonstrated thus far. The TreP pathway is the reversible hydrolysis of trehalose in presence of inorganic phosphate by the trehalose phosphorylase enzyme (TreP). The reversible TreT pathway consists of the formation of trehalose and ADP from ADP-glucose and glucose by the trehalose glycosyltransferring synthase enzyme (TreT). Ac, Archea; B, Bacteria; F, Fungi; P, Plants; Ap, Arthropods; Pr, Protists. * Trehalase is present in all kingdoms, including mammals.

Trehalose can be synthesized via different metabolic pathways [2], sometimes coexisting, with both shared and specific functions relevant to pathogenicity (Table 1). Of the three potential trehalose biosynthesis pathways differently contributing to *Mycobacterium tuberculosis* virulence, the OtsAB pathway (Figure 1) is the dominant one [3]: deletion of OtsA results in severe growth defects, whereas the OtsB gene is essential for viability. Of the two additional pathways, the TreYZ pathway and the TreS pathway, only the latter seems necessary for late development of chronic infections in mice, while absence of the first doesn't cause any apparent defect in microbial growth or virulence.

**Table 1:** Divergence in the relevance of the three main trehalose biosynthetic pathways in the viability and pathogenicity of prokaryotes.

	PATHWAY		
ORGANISM	OtsAB/TPS-TPP	TreY/Z	TreS
*M. tuberculosis* [3]	Dominant OtsB2: essential OtsB1: pseudogene Deletion of OtsA leads to reduced virulence in mice	No role in cell viability *in vitro* and *in vivo*	Role in prolonged infection
*M. bovis* [3]	OtsB2 and OtsA are essential enzymes		
*M. smegmatis* [13]	Redundancy between the three pathways: single deletions have no apparent phenotypes, while triple deletions result in growth inhibition at high temperatures		
*M. leprae* [34]	Unique intact pathway	pseudogene	pseudogene
*Corynebacterium glutamicum* [14], [15]	Contributes to glycolipids synthesis together with the TreYZ pathway	Dominant pathway in osmotic conditions	Contributes to trehalose degradation
*E. coli* [35]	Sole pathway	none	none
*Salmonella enterica* [36]	Sole pathway involved in environmental survival but not in virulence)	none	none
*Pseudomonas syringae* [4]	none	Both pathways are required for trehalose synthesis and depend on each other (the TreS-mediated trehalose synthesis may depend on maltose generated by the TreY/TreZ pathway)	

Only the TreS and TreYZ pathways are present in the plant pathogen *Pseudomonas syringae*, and their absence leads to reduced survival on host tomato leaves [4], an effect that may be attributed, at least in part, to a fitness defect of the corresponding mutants in low-water environments. Inactivation of the same two pathways reduces the pathogenicity of *Pseudomonas aeruginosa* against plants, restored by co-inoculation of exogenous trehalose, but not against worms, insects, and mice [5].

In fungal pathogens, trehalose is mainly synthesized via the TPS/TPP pathway (Figure 1). Originally demonstrated in *Candida albicans* [6], the abolishment of trehalose production has a significant negative impact on the *in vivo* survival of several plant and human pathogens, including *Magnaporthe oryzae* [7], *Stagonospora nodorum* [8], *Cryptococcus neoformans* [9], and *Cryptococcus gattii* [10]. *Aspergillus fumigatus*, the causative agent of invasive pulmonary disease in immunocompromised patients, represents an exception to this pattern. In this organism, disruption of the two trehalose 6-phosphate synthase genes, *tpsA* and *tpsB*, surprisingly leads to higher resistance to phagocytosis and hypervirulence in mice, despite the complete absence of trehalose accumulation and severely reduced fitness of these mutants [11]. Conversely, deletion of the trehalose 6-phosphate phosphatase *OrlA* (Figure 1) results in avirulence, despite the absence of growth defects *in vitro* [12].

## Trehalose Is Associated with Several Biological Processes: Sporulation, Germination, Metabolism, and Morphogenesis

Trehalose and trehalose biosynthetic enzymes have emerged as essential players in virulence-associated phenotypes. Interestingly, the hierarchy of trehalose biosynthetic pathways appears to be species-specific. Contrary to the case of *M. tuberculosis*, described above, in the closely related *M. smegmatis* the OtsAB, TreYZ, and TreS pathways show a great level of redundancy [13], whereas osmotically regulated trehalose synthesis in *Corynebacterium glutamicum* is predominantly mediated by the TreYZ pathway (Table 1) [14], [15]. Independently from these differences in metabolic routes, however, impairment in trehalose production results in severe growth defects in all mentioned species. In *Pseudomonas syringae*, trehalose accumulation is abolished in mutants of either producing pathways, with consequences on growth in hyperosmotic but not in stress-free conditions.

Impairment of trehalose homeostasis results in increased susceptibility to oxidative stress in *Aspergillus nidulans* [16], defects in melanin synthesis, capsule production, mating, and cell wall integrity in *Cryptococcus gattii* but not in *C. neoformans* [9], [10], and in poor sporulation in *S. nodorum* and *M. oryzae* [7], [8]. In the latter species, Tps1 governs the process of carbon catabolite repression, via glucose 6-phosphate sensing, favouring the utilization of glucose over other carbon sources [17]. Multiple functions for the trehalose 6-phosphate synthase Tps1 were uncovered in *M. oryzae*. Besides trehalose biosynthesis, Tps1 regulates nitrogen source utilization, hence contributing to adaptation of the pathogen to the plant host [18]. Recently, a similar process has been identified in *P. aeruginosa*, in which trehalose itself was shown to promote the acquisition of nitrogen-containing nutrients upon infection of plants [5]. *M. oryzae* Tps1 also controls transcriptional effectors linked to virulence factors via NADPH-binding during appressorium-mediated rice infection [19].

In *A. fumigatus*, disruption of the two trehalose 6-phosphate synthase genes, *tpsA* and *tpsB*, leads to the complete absence of trehalose accumulation, and to defects in spore germination, growth at high temperature, and susceptibility to oxidative stress [11]. Deletion of the trehalose 6-phosphate phosphatase gene *OrlA* in this organism results in unaltered levels of trehalose and increased levels of trehalose 6-phosphate. Two putative trehalose phosphorylases (TreP) (Figure 1) were found to be encoded by the genome of *A. fumigatus* [12]. Functionality of the TreP pathway in this organism might explain the unexpected accumulation of trehalose and trehalose 6-phosphate in *orlA* mutants. However, this hypothesis is awaiting experimental validation.

In *C. albicans*, absence of a functional trehalose synthesis machinery causes defects in the yeast to hyphal transition [6], whereas high levels of trehalose correlate with deficient Hsp90-dependent cell elongation in response to elevated temperature [20]. Trehalose accumulation is induced by addition of amphotericin B in *C. albicans* [21]. This ergosterol-targeting antifungal promotes trehalose synthesis activity and deactivation of the neutral trehalase; however, it is not known whether high trehalose can reduce the fungal susceptibility to amphotericin B.

## Fluctuations and Turnover of Trehalose Content during Infection Processes: It's All about Timing

Trehalose is essential at certain stages of development or in certain environmental conditions, and its abundance varies from undetectable levels to up to 10% of dry weight in response to stress, in late phases of the life cycle, or during the infection process. Increased trehalose contents were suggested to indicate adaptation of microorganisms to adverse or stressful conditions. Recycling of released trehalose is essential for virulence of *M. tuberculosis* [22]. The trehalose moiety of the glycolipid trehalose monomycolate is released extracellularly, and its re-uptake, mediated via an ABC transporter, is essential to virulence. In *Salmonella enterica*, mutants with a defect in trehalose biosynthesis are still virulent. Interestingly, however, failure to import the compatible solute glycine betaine in Δ*opuA-D* mutants leads to a strong increase in trehalose biosynthesis under stress conditions, ultimately resulting in increased tolerance to stress conditions mimicking the host innate immunity. The trehalose-dependent higher proficiency of Δ*opuA-D* mutants to colonize host tissues, as compared to the wild type, indicate that the OpuA-D transporter represses trehalose biosynthesis, therefore limiting virulence in favour of chronic infections [23]. Thus, while the absence of trehalose has no known effect *in vivo*, high levels of the disaccharide may promote more aggressive infections. In *P. aeruginosa*, trehalose, like other metabolites such as amino acids and acetate, is differentially abundant during the evolution of lung infections in patients affected by cystic fibrosis [24]. In *Klebsiella pneumoniae*, expression of the *treB* and *treC* genes, involved in trehalose uptake and hydrolysis, respectively (Figure 1), is induced during early stages of biofilm formation, and the absence of either gene impairs biofilm development [25]. Proper trehalose utilization and catabolism into glucose and glucose 6-phosphate is required for capsule formation, known to play a role in biofilm formation *in vitro* as well as in a murine model of gastrointestinal tract colonization.

In fungi, while synthesis of trehalose is required for proficient initial plant infection by *M. oryzae*, trehalose mobilization plays a role in the colonization step [7]. Deletion of the neutral trehalase Nth1 in *C. gattii* has no effect on virulence [10], while in *C. albicans*, deletion of the cell wall–associated acid trehalase, but not of the neutral trehalase, has a negative impact on morphogenesis and virulence in mice [26]. Trehalose mobilization is required for efficient growth resumption in favourable conditions, and this may also be associated with the use of trehalose as a carbon source. Trehalose levels also vary in *C. albicans* biofilms, where its content increases dramatically in the first six hours of biofilm formation, followed by a decline in mature biofilms [27]. The exact role of these fluctuations remains elusive, however it is tempting to speculate on an antioxidant function of trehalose in the early stages of biofilm formation, and a role as energy supply in mature structures. Alternatively, it is also possible that in the initial phases of biofilm formation, high trehalose levels may prevent filamentation [20] so yeast cells can colonize the whole substrate, and that at later stages, lower trehalose levels allow filament formation. The decline in trehalose at later stages may also indicate a shift in metabolic flux as more UDP-glucose is likely required for β-glucan synthesis during matrix formation, resulting in less substrate for trehalose biosynthesis.

## Trehalose and Lipids: A Unique Collaboration

A wealth of information describes the unique relationship between trehalose and mycolic acids in Mycobacteria and the phylogenetically related Corynebacteria [28], [29]. Trehalose and mycolic acid form trehalose 6,6-dimycolate (TDM), or cord factor, which is the most abundant glycolipid in Mycobacteria. Involvement of TDM in several aspects of pathogenicity has been demonstrated, including protection against phagocytes' killing, evasion from the immune response, and reduction of antibiotic effectiveness. Tissue damage and necrosis caused by its association with host lipids were also reported. Recent findings point toward the recognition of TDM by the host innate immune cells via the Mincle C-type lectin receptor, which also recognizes other pathogens such as *C. albicans*, *Malasezzia* spp., and *Fonsecaea pedrosoi* in a TDM-independent manner [30]. Yet, the ligand activity of TDM requires both the sugar and lipid moieties, as both components separately do not activate Mincle-expressing cells [30]. Bacterial cell wall–associated lipids, and in particular TDM, are also important for the infection process in the Gram-positive bacterium *Nocardia brasiliensis*. Absence of TDM and other hydrophobic compounds abolishes the infection without affecting the pathogen's viability [31].

Besides TDM, trehalose is the precursor of several metabolites and cell-wall glycolipids, the so-called “trehalome” of Mycobacteria. Detection and visualization of the cell surface trehalome were recently described [32]. Probing the trehalome in a pathway-targeted manner, including the recycling pathway, is now possible in several mycobacterial species, including *M. tuberculosis*. This represents a promising perspective for the study of the dynamics and trafficking of the mycobacterial trehalome during the infection process.

What does the future hold for trehalose and its synthesis, and in particular what does it hold for the field of antimicrobial research? No doubt that the beat goes on! The discovery of a novel pathway from trehalose to glycogen and α-glucans in *M. tuberculosis* leads the way. The GlgE enzyme is validated as a drug target candidate by the combination of its essentiality in a synthetic lethal pathway, and by the absence of GlgE homologs in humans and commensal gut microflora [33]. Despite or perhaps due to its versatility, trehalose stands out as a major player in the lifestyle of pathogens. The challenge, however, remains in the identification of molecular links between trehalose or its metabolism and pathways regulating the ability of pathogens to infect their host and/or to hide from the host.
